# Influence of sedation on morbidity and mortality in the intensive care unit

**DOI:** 10.1590/S1516-31802004000100003

**Published:** 2004-01-08

**Authors:** Junior Geraldo Rolim Rodrigues, José Luiz Gomes do Amaral

**Keywords:** Intensive care unit, Complications, APACHE, Mortality, Complicações, Mortalidade hospitalar, APACHE, Unidades de terapia intensiva, Cuidados intensivos

## Abstract

**CONTEXT:**

Although 30 to 50% of hospitalized patients in a critical care unit are under sedation, there is sparse data on the impact of sedation on morbidity and case-fatality rates in Brazil. Sedation is associated with higher risks of infection and death rate among patients. However, it is difficult to assess the clinical impact of sedation.

**OBJECTIVE:**

To evaluate the impact of sedation on the incidence of nosocomial infection and all-cause deaths at a critical care unit.

**TYPE OF STUDY:**

Prospective study.

**SETTING:**

Tertiary-care teaching hospital.

**PARTICIPANTS:**

After the exclusion of patients hospitalized for less than 24 hours, 307 patients were assigned to two groups, considering their states of sedation. After confirmation of heterogeneity in relation to the Acute Physiology and Chronic Health Evaluation (APACHE II) prognostic system, 97 sedated and 97 non-sedated patients were matched in relation to this severity index.

**MAIN MEASUREMENTS:**

Impact of sedation on deep venous thrombosis, incidence of decubital eschars, presence of infection, mortality and length of hospital stay.

**RESULTS:**

There was no difference in the incidence of deep venous thrombosis between the sedated and non-sedated groups, while the frequency of decubital eschars was significantly higher among sedated patients (p = 0.03). Infection was detected in 45.4% of patients under sedation and 21.6% of patients not under sedation (p = 0.006). Mortality for patients that did not receive any kind of sedative was 20.6% and, for those that were sedated during hospitalization, the rate was 52.6% (p < 0.0001). The sedated patients had longer hospitalization (11 vs. 4 days) (p < 0.0001).

**CONCLUSION:**

We concluded that sedation is associated with higher infection risk and case-fatality rate, and longer hospital stay.

## RESUMO

**CONTEXTO:**

Embora cerca de 30% a 50% dos pacientes hospitalizados em unidades de terapia intensiva (UTI) recebam algum tipo de sedativo, existe escassez de informações sobre efeitos adversos desta prática, especialmente no Brasil. Estes efeitos podem ser significantes e o uso de sedativos é associado a elevação de infecção e mortalidade, mesmo sendo difícil avaliar o impacto clínico deste procedimento.

**OBJETIVO:**

Avaliar o impacto da sedação sobre incidência de complicações e mortalidade em doentes graves durante internação em unidade de terapia intensiva.

**TIPO DE ESTUDO:**

Estudo prospectivo.

**LOCAL:**

Unidade de Terapia Intensiva Cirúrgica da Universidade Federal de São Paulo — Escola Paulista de Medicina.

**PARTICIPANTES:**

Após excluídos pacientes que permaneceram menos de 24 horas ou sem exames indispensáveis para o cálculo do índice de gravidade (APACHE II), restaram 307 pacientes. Estes foram divididos em dois grupos: Grupo Sedado e Grupo Não Sedado. Constatada heterogeneidade com relação ao APACHE II, foram pareados 97 sedados e 97 não sedados com idênticos índices de gravidade.

**VARIÁVEIS ESTUDADAS:**

Impacto da sedação e das técnicas sobre a mortalidade, tempo de internação, além da incidência de escara de decúbito ou pressão, trombose venosa profunda e infecção.

**RESULTADOS:**

Não houve diferença na incidência de trombose venosa profunda, entre os grupos Sedado e Não Sedado, enquanto que escara de decúbito foi significativamente maior nos sedados (p = 0,03). Infecção foi detectada em 45,4% dos pacientes com sedação e em 21,6% dos pacientes sem sedação (p = 0,006). A mortalidade para os pacientes que não receberam qualquer tipo de sedativo foi de 20,6% e, para aqueles que foram sedados durante a internação, foi de 52,6% (p < 0,0001).

**CONCLUSÕES:** Conclui-se que a sedação está associada a maior duração da internação, morbidade e mortalidade significativas. Apesar da intensidade das associações encontradas, não é possível estabelecer relação causal entre sedação e mortalidade.

## INTRODUCTION

Over the last few decades, intensive care practice has been much influenced by scientific and technological progress. New generations of equipment and drugs have become constant achievements for intensive care specialists. Through the use of sophisticated monitoring and therapeutic resources, these specialists try to qualify, quantify and control a number of biological phenomena.

Among the indications for sedation is the institution and maintenance of artificial ventilation. Many interventions require sedation, including such uncomfortable or painful procedures as tracheal intubation. Another possible cause of anxiety requiring sedation is the relative immobility needed by some sensitive monitoring systems, which demands patience and collaboration beyond the limits imposed by the illness. The behavior known as "intensive care unit" can range from gentle agitation to intense aggressiveness and violence that requires immediate physical containment and the use of sedatives. In patients with coronary disease or acute respiratory insufficiency, agitation can harm the clinical evolution. The importance of sedation for the treatment of critical patients has become obvious.

Even so, it is difficult to evaluate the clinical impact of this procedure. Although about 30 to 50% of hospitalized patients receive some sort of sedation, which usually includes opiates alone (37%) or in association with benzodiazepines (60%),^1-3^ this can lead to significant side effects. Sedation has been associated with higher risk of infection and higher case-fatality rates.^1^

The main aim of this study, considering the shortage of information about sedation in the intensive care units of Brazil, was to determine the impact of sedation on the length of hospital stay, the incidence of complications like decubital eschar, deep venous thrombosis and infections, and the case-fatality rate.

## METHODS

Over an 11-month period, 307 patients (182 men and 125 women) hospitalized in an intensive care unit at a tertiary-care teaching hospital were evaluated, after the exclusion of patients hospitalized for less than 24 hours or those whose examination was inadequate for the calculation of the severity index (Acute Physiology and Chronic Health Evaluation — APACHE II prognostic system). The APACHE II index confirmed the heterogeneity of these 307 patients in relation to the initial severity of their condition.

After matching according to the APACHE II severity index and in relation to sex and age, 97 non-sedated patients and 97 sedated patients were selected, thereby forming two groups: the non-sedated group for patients that did not receive any kind of sedative, and the sedated group for those that were sedated during hospitalization.

Analysis was made of the incidence of decubital eschars, deep vein thrombosis and infections, and also the influence of sedation on the length of hospital stay and mortality. A diagnosis of decubital eschars was made when areas of ulceration or necrosis were present in anatomical regions that were subjected to more intense pressure (calcaneum and sacrum). Cases of suspected deep vein thrombosis were confirmed by means of venous Doppler and/or angiography. The presence of infection was determined from the clinical signals and culturing that indicated outbreaks of infection. Analysis was also made of whether the different sedation procedures used had an influence on the incidence of complications.

For statistical analysis, the Mann-Whitney test^4^ for independent groups was applied, comparing the sedated and non-sedated groups according to APACHE II prognostic system results, length of hospital stay and age in years. This test was performed using an approximation to the normal curve (z statistics). The chisquared test^4^ was utilized for association tables, comparing the sedated and non-sedated groups in relation to the characteristics studied. The Fisher exact test was applied, with the use of the Cochran restrictions.^4^ For all tests, the level of hypothesis rejection was set at 0.05 or 5%.

## RESULTS

There were 192 patients that did not receive any kind of sedative, while 115 patients received some kind of sedative. The sedated and non-sedated groups were heterogeneous in relation to initial severity and it was therefore decided to form matched pairs of cases with identical initial severity. The result from this was two matched groups of 97 patients in each. In the sedated group, there were 64 men (66%) and 33 women (34%), with a median age of 50 years. In the non-sedated group with identical severity indexes, there were 60 men (62%) and 37 women (38%), with a median age of 53 years. These data are all shown in Table 1.

**Table 1:** Comparison of sedated and non-sedated patients, with and without APACHE II prognostic system matching in relation to age, sex, length of hospital stay (in days), case-fatality rate, incidence of infection, decubital eschars, deep venous thrombosis and main diagnosis

Group	Sedated	Non-sedated
Average APACHE II (µ)	18*	15
Average APACHE II after matching (µ)	16	16
Average age (years)	50	53
Male (%)	66	62
Female (%)	34	37
Length of hospital stay (days)	11*	04
Case-fatality rate (%)	52.6*	20.6
Infection (%)	45.4*	21.6
Decubital eschars (%)	7.2*	1
Deep venous thrombosis (%)	1	1
Main diagnosis	Acute respiratory distress syndrome	Major surgery postoperative
	Multiple trauma	Sepsis

* *Asterisk indicates statistical significance.*

Although matched for the same initial severity, the sedated patients had longer hospital stays than the non-sedated patients (11 vs. 4 days) (p < 0.0001) (Table 1).

The higher frequency of infections among sedated patients (45.4% vs. 21.6%, p = 0.0063) was statistically significant (Table 1). The same was found in relation to mortality, which was much more frequent in the sedated group (52.6 vs. 20.6%) (p < 0.0001), as shown in Figure 3. This may have been due to the worse evolution of such patients.

The incidence of decubital eschars among the patients in the sedated group was 7.2% while it was 1% for the non-sedated group (p = 0.0323, Table 1).

It was not possible to verify a difference between the groups in relation to the incidence of deep vein thrombosis (p = 0.7512) (Table 1).

## DISCUSSION

Sedation has been implicated as a potential factor for mortality. In the present study, there was a case-fatality rate of 52.6% among sedated and 20.6% among non-sedated patients. The case-fatality rate was directly correlatable with the severity of the disease, and this can be explained by the limitations of APACHE index sensitivity. The in-hospital infection ratio was 45.4% for the sedated and 21.6% for the non-sedated group. There was no variation in the incidence of deep vein thrombosis between the sedated and non-sedated patients, appearing in both groups at around 1%. However, there was a difference between the groups in relation to the incidence of decubital eschars (7.2% and 1%, in the sedated and non-sedated patients).

Although Ramsay et al.^5^ did not observe significant side-effects in patients under continuous infusion of sedatives, it is known that sedation is not risk-free. Complications are likely to arise mainly during extended treatment.^6^ In extended sedation, immobility is associated with negative nitrogen balance^1^ and increased incidence of lesions in nerves or skin due to the decubitus position.^7^ In the present data, we did not observe any difference in the incidence of deep vein thrombosis between the two groups, and this may have been because of the intensive nursing care. However, the subjacent disease in such patients determines hospitalization and extended periods of restriction to bed. Thus, it is difficult to blame sedation for such complications.

There are few data on mortality and sedation-related complications. Probably the only research that related sedative administration to increased mortality was a classic study on the impact of sedative agents on very critical patients, in which the authors found mortality of around 28% among patients submitted to opiates and benzodiazepines, which increased to 77% when associated with etomidate.^8^ The authors also observed that mortality increased according to the severity of the disease (as estimated by ISS, the Injury Severity Score). The hypnotic agent etomidate, when used in continuous sedation, has been associated with significant increases in mortality.^8-10^ However, such observations were not confirmed by Döenicke.^11^ In that study, none of the patients in the sedated group received etomidate as a sedative, even though that was the group with the highest mortality. This probably occurred because of the patients' heterogeneity and their worse evolution.

Sedation is equally associated with a higher risk of infection.^12-14^ However, the immunological state is usually a function of the underlying disease or trauma. There is some evidence showing the possible interference of sedative agents in the body's defenses (usually among critical patients), perhaps suppressing polymorphonuclear functions^2^ or acting as a vector for the growth and transmission of microbial agents.^13^

This may have led to indirect sedative action on the immunological system or may have promoted the growth of pathogenic organisms that would be transmitted to the almost 46% of the patients that presented infection. In this group, the causal relationship of these results is not clear because the sedation was deeper and more prolonged and the affections were more serious, even when the APACHE II prognostic system indicated uniformity. This probably occurs because the severity index has limitations and does not clearly denote some situations of increased severity, since it is performed within the first 24 hours of hospitalization.

The Acute Physiology and Chronic Health Evaluation II (APACHE II prognostic system) is obtained through the evaluation of 12 clinical patterns. After such evaluation the patients are classified into four clinical statuses, according to the patients' physical status. This classification allows the prediction of the patients' evolution. The greatest limitation to the APACHE II score is that it must be done within the first 24 hours after admission of the patient to the Intensive Care Unit, and it does not take into account any underlying disease complications and their evolution.^15^

We also verified that mortality increases when there is a higher APACHE II. Even when matching the patients with identical APACHE II indexes or using a risk of death derived from this, the mortality continued to be higher in the sedated group. Once more, it was seen that a more critical group submitted to a longer period of sedation was selected, even with matched APACHE II indexes. In this group, the patients were possibly submitted to more mechanical ventilation procedures, because the initial nosocomial entities were associated with lung diseases, especially acute respiratory distress syndrome, which increases mortality to higher rates than what would be expected from the majority of diseases that lead the patient to intensive therapy. In such cases, a higher degree of sedation is necessary in order to adapt the patient to uncomfortable artificial ventilation procedures, so as to achieve better blood oxygenation, which had deteriorated because of the disease. However, larger sedative infusions are necessary because of the severity of symptoms caused by the clinical state, thus increasing the mortality rate, as shown in this study. Nonetheless, a direct relationship cannot be established, since the patients with acute respiratory distress syndrome still presented the same low APACHE II seen initially, throughout their hospitalization. However, sedation in these cases was increased and the possibility of lethal outcomes became higher. Therefore, it is not possible to establish a clear cause-effect relationship between sedation and increased infection and mortality, even with the finding of statistical significance.

Despite the difficulty in quantifying the benefits, and the shortage of information, sedation has wide application in this field.^1, 16, 17^ Most of the patients in intensive therapy need analgesia, sedation or both, and these are administered in 30 to 50% of seriously ill patients.^1^

In recent national survey carried out by the Brazilian Intensive Care Association,^18^ fentanyl was the analgesic agent most often used by Brazilian specialists. The task force from the American College of Critical Care Medicine and the Society of Critical Care Medicine^19, 20^ has recommended morphine as the first choice, especially due to its low cost. The same recommendations indicated fentanyl as the first choice,^20^ especially in cases of histamine release and hemodynamic instability.^19^ The Brazilian Intensive Care Association task force recommended morphine and fentanyl equally, following meetings held in 1997 and 1999, as the chosen first level drug (procedures or drugs recommended from scientific evidence and enough clinic experience in Brazil).^21,22^

In fact, as demonstrated in the survey by the Brazilian Intensive Care Association, the most commonly used techniques in the world include opiates alone or in association with benzodiazepines. Fentanyl or morphine in association with midazolam or propofol are the most frequently recommended therapy^18-22^ and constitute almost 80% of all the sedatives used in Brazil. Neuromuscular blockers provide complementary sedation for artificially ventilated patients in about 22.7% of the cases.

These data support the need for careful monitoring of the use of sedatives in intensive care that might have a potential for influencing the immune status of critically ill patients. Nevertheless, the complexity of treatments and the heterogeneity of patients have not allowed evaluation of the overall impact of sedatives on critically ill patients. At the same time, these data have indicated that sedation and analgesia are essential aspects in the treatment of patients within intensive care units. The low accuracy of the APACHE II index and the heterogeneity of patients have limited the results from this study. Evaluation of the influence of sedatives on morbidity and case-fatality rates among critically ill patients is a new field of research. Further studies are therefore needed in order to determine their real impact.

## CONCLUSION

The results have indicated that sedation is associated with longer hospital stays and higher risk of infection. Despite the intensity of the associations found, it is not possible to establish a causal relationship between sedation and the case-fatality rate.
